# Lutathera^®^: The First FDA- and EMA-Approved Radiopharmaceutical for Peptide Receptor Radionuclide Therapy

**DOI:** 10.3390/ph12030114

**Published:** 2019-07-29

**Authors:** Ute Hennrich, Klaus Kopka

**Affiliations:** 1 German Cancer Research Center (DKFZ), Division of Radiopharmaceutical Chemistry, Im Neuenheimer Feld 280, 69120 Heidelberg, Germany; 2 German Cancer Consortium (DKTK), Im Neuenheimer Feld 280, 69120 Heidelberg, Germany

**Keywords:** Lutathera^®^, [^177^Lu]Lu-DOTA-TATE, thera(g)nostics, peptide receptor radionuclide therapy (PRRT), somatostatin receptor (SSTR), neuroendocrine tumors (NET)

## Abstract

As the first radiopharmaceutical for Peptide Receptor Radionuclide Therapy (PRRT), Lutathera^®^ was approved by the EMA in 2017 and the FDA in 2018 for the treatment of somatostatin receptor (SSTR) positive gastroenteropancreatic neuroendocrine tumors. Using the concept of PRRT, Lutathera^®^ combines the radionuclide ^177^Lu with the somatostatin analogue DOTA-TATE, thus delivering ionizing radiation specifically to tumor cells expressing somatostatin receptors. As a result, DNA single- and double-strand breaks are provoked, in case of double-strand breaks leading to cell death of the tumor and its SSTR-positive lesions.

## 1. Introduction

In January 2018 (26 January 2018), Lutathera^®^ ([^177^Lu]Lu-DOTA-TATE (for rules on nomenclature, see [1,2])) was approved by the FDA [3] for the treatment of somatostatin receptor (SSTR) positive gastroenteropancreatic neuroendocrine tumors. The approval of Lutathera^®^ in Europe was granted in September 2017 (26 September 2017) by the EMA [4]. Recently (9 January 2019), this breakthrough thera(g)nostic radiopharmaceutical also received approval by Canada Health [5]. The holder of the marketing authorizations is Advanced Accelerators Applications S.A. (AAA, a Novartis company).

The use of a thera(g)nostic approach in nuclear medicine has gained ever more importance in the last few years and is the epitome of personalized medicine [6]. As a first step in the thera(g)nostic concept, a target molecule labeled with a diagnostic radionuclide is used for quantitative imaging of a tumor imaging biomarker, using positron emission tomography (PET) or single photon emission computed tomography (SPECT). When it is demonstrated that, with this targeted molecule, a tumoricidal radiation absorbed dose can be delivered to tumor and metastases, as a second step, the administration of the same or a similar target molecule labeled with a therapeutic radionuclide will be conducted. Such a class of thera(g)nostic tracers is, for example, [^177^Lu]Lu-DOTA-TATE for endoradiotherapy, together with its diagnostic partner [^68^Ga]Ga-DOTA-TATE (in the United States, NETSPOT^TM^) or [^68^Ga]Ga-DOTA-TOC (DOTA-(D-Phe^1^,Tyr^3^)-octreotide, in the European Union, SomaKit TOC^®^). 

The therapeutic tracer [^177^Lu]Lu-DOTA-TATE uses the concept of Peptide Receptor Radionuclide Therapy (PRRT), a form of endoradiotherapy: By combining a suitable radionuclide with a peptide that specifically binds to a peptide receptor, which is upregulated to a high concentration (determined and expressed as B_max_) on tumor cells and at low physiological levels on normal tissues, cytotoxic radiation is delivered to the tumor. The specific peptide is conjugated with an appropriate chelator for complexing the radionuclide. Essential properties of the peptide carrier are, i. a., high binding affinity for the target receptor, in vivo stability, and a fast clearance from blood and other non-target tissue. Upon binding of the radiolabeled peptide to the receptor, ideally, an internalization of the compound takes place, facilitating a long retention in the tumor cells and the emission of ionizing radiation from the bound radionuclide to selectively destroy the peptide receptor positive tumor cells [7]. Therapeutic radionuclides are either particle emitters (α or β) or they decay by electron capture (EC), leading to the emission of Auger electrons and gamma rays. A prerequisite is a high linear energy transfer (LET) of the α particle or electron to locally deliver cytotoxic ionizing radiation to the tumor cells [7]. The choice of the radionuclide for endoradiotherapy depends mostly on the size of the tumor.

Neuroendocrine tumors (NETs) belong to a group of tumors originating from neuroendocrine cells distributed throughout the human body. Approximately two thirds of NETs derive from the gastrointestinal system and represent the group of gastroenteropancreatic tumors (GEP-NETs) [8]. Most NETs show relatively high expression levels of SSTRs, which offers the possibility of imaging and therapy using radiolabeled somatostatin analogues [9]. There are five subtypes of SSTRs, SSTR1 to SSTR5. Most commonly expressed in NETs are SSTR2 and SSTR5, but there is a considerable variability in expression of SSTR subtypes in different tumor types [7]. Radiolabeled somatostatin analogues, which exhibit a high affinity for SSTRs, are ideal candidates for PRRT because by internalization of the receptor–peptide complex by endocytosis, the radiopeptide is retained in the receptor-expressing tumor cells, and due to its relatively low molecular weight, it is rapidly cleared from blood [7]. The development of these radiolabeled somatostatin ligands was a very long scientific journey that started with the first description of somatostatin more than 45 years ago [10]. A number of diagnostic as well as therapeutic somatostatin ligands have been developed over the years and some of them are summarized in a review by Kwekkeboom et al. [11]. The first radiolabeled SST analog used for imaging was [^123^I, Tyr^3^]octreotide in 1989 [12], while early therapeutic studies used [^111^In]In-DTPA-octreotide in the mid to late 1990s, since other chelated somatostatin ligands were not available [11]. Further developments lead to [^177^Lu]Lu-DOTA-TATE, a very promising candidate for endoradiotherapy, which was first described in 1999 [13]. First clinical studies started in 2000 in Rotterdam (The Netherlands) [14]. A large clinical study including 1214 patients was the Erasmus phase-I/-II study, a monocentric single arm open label study (Rotterdam, The Netherlands) to evaluate the efficacy of [^177^Lu]Lu-DOTA-TATE [8,15]. Because of the promising results, the NETTER-1 study, a phase III study with 229 patients, was initiated in 2011 [16]. This study was a multicenter, stratified, open, randomized, comparator-controlled, parallel-group phase-III study comparing the treatment of patients with [^177^Lu]Lu-DOTA-TATE plus octreotide LAR (long acting release, for symptoms control, 30 mg) to the treatment with a high substance dose of octreotide LAR (60 mg) [8]. Comparison of progression free survival (PFS) was the primary objective and the primary efficacy endpoint of the trial. There was a highly significant difference (*p* < 0.0001) between the two groups: At 20 months, 65% of patients in the [^177^Lu]Lu-DOTA-TATE group were living and free of progression, compared to only 11% in the control group [8,13]. The median overall survival was 27.4 months in the octreotide LAR arm, compared to the [^177^Lu]Lu-DOTA-TATE group where this end point was still not reached after 42 months [8]. The median PFS was 8.5 months for the control group, compared to the [^177^Lu]Lu-DOTA-TATE group in which median PFS was not reached [8]. Last, but not least, patients in the [^177^Lu]Lu-DOTA-TATE group had a significantly higher objective response rate, compared to the control group (18% vs. 3%; *p* < 0.001) [8]. It could also be demonstrated that treatment with [^177^Lu]Lu-DOTA-TATE was safe and well tolerated [8].

## 2. Chemical Overview

### 2.1. Names and Structure

**Figure 1:**
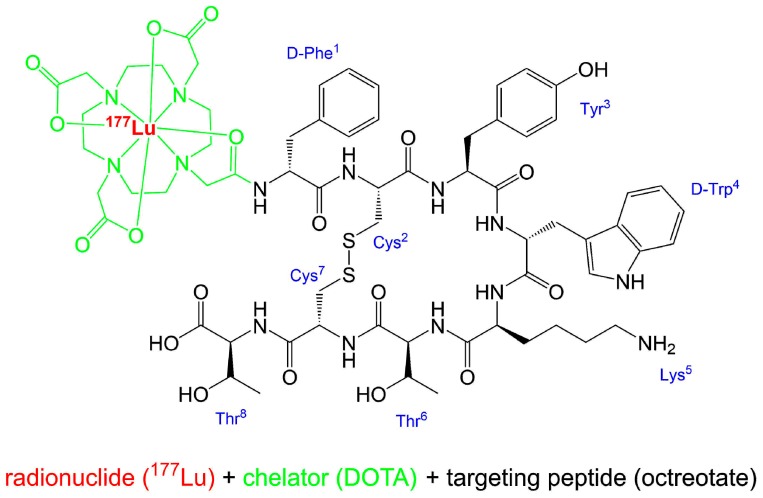
Structure of [^177^Lu]Lu-DOTA-TATE (Lutathera^®^). Simplified writing of complexation [18].

[^177^Lu]Lu-DOTA-TATE (Figure 1) is the active ingredient of Lutathera^®^, which is commercially available by Advanced Accelerator Applications S.A. (AAA, a Novartis company). Other names for [^177^Lu]Lu-DOTA-TATE are ^177^Lu-Oxodothreotide or [^177^Lu]Lu-DOTA-(Tyr^3^)-octreotate and its IUPAC name is:

[^177^Lu]lutetium-*N*-[(4,7,10-tricarboxymethyl-1,4,7,10-tetraazacyclododec-1-yl)acetyl]-*D*-phenylalanyl-*L*-cysteinyl-*L*-tyrosyl-*D*-tryptophanyl-*L*-lysyl-*L*-threoninyl-*L*-cysteinyl-*L*-threonin-cyclic(2–7)disulfide. 

The radionuclide ^177^Lu is complexed by the bifunctional chelator DOTA, which acts as an octadentate chelator [17] and is thus bound to the somatostatin affine peptide (Tyr^3^)-octreotate. 

### 2.2. Lutetium-177

The radionuclide lutetium-177 (^177^Lu) decays with a half-life of 6.65 days by emission of electrons to the stable isotope haffnium-177 (^177^Hf) [19]. While the maximal β^−^ energy is 498.3 keV (79.4%), the average β^−^ energy is 134.2 keV. This results in a maximal tissue penetration of the β-particles of 2.2 mm with an average penetration range of 0.67 mm [8]. Therefore, ^177^Lu is a so called short-range β particle emitter [20] and while the penetration is sufficient to kill a peptide receptor positive targeted tumor cell, it shows limited effects on neighboring normal tissue. During decay, additionally to β^−^ emission, low energy γ-rays are emitted, e.g., with energies of 112.9 keV (6.17%) and 208.4 keV (10.4%) [19]. These γ-rays can be used for imaging of patients after the intravenous injection (i. v.) of the sterile [^177^Lu]Lu-DOTA-TATE injection solution, using scintigraphy or SPECT. 

For comparison, another widely used trivalent radiometal for enodradiotherapy is yttrium−90 (^90^Y) with a half life of 2.7 days, an average β^-^ energy of 935 keV and a mean particle penetration range of 3.9 mm [20]. This radionuclide is a long-range pure β particle emitter and may be advantageous in comparison to ^177^Lu when dealing with larger tumors. Since ^90^Y does not emit suitable γ-rays, a detection of the radiopharmaceutical after administration is difficult. It is possible by Bremsstrahlung scintigraphy or PET using SPECT or PET scanners [21,22], but this yields only low quality images in comparison to state-of-the-art SPECT and PET images where typical SPECT or PET radionuclides are used.

### 2.3. Manufacturing and Quality Criteria

[^177^Lu]Lu-DOTA-TATE is produced in a GMP (Good Manufacturing Practice)-compliant automated radiosynthesis by reacting n.c.a. (no-carrier added) [^177^Lu]LuCl_3_ with DOTA-TATE under suitable conditions [8]. A detailed description of a typical automated radiosynthesis of [^177^Lu]Lu-DOTA-TATE, including quality control, is given in a publication by Aslani et al. [23]. Following the reaction, the product is purified, formulated, and sterile filtered [8]. The radiochemical purity and other parameters of the final sterile product solution are controlled [24,25]. Only if all quality criteria are passed, the product will be released for patient application. AAA production sites for Lutathera^®^ are in Colleretto Giacosa (Italy) and Meldola (Italy). Due to the half life of ^177^Lu, a global distribution of the radiotracer is feasible. The finished product manufactured by AAA is dispensed into ready-to-use single dosages containing 7.4 GBq ± 10% of Lutathera^®^ in aqueous solution (20.5–25 mL) at the time of administration to the patient [8,26]. The injection solution is clear and colorless to slightly yellow with a pH value of 4.5–8.5 [27] and it has an activity concentration of 370 MBq/mL at calibration time (calibration time corresponds to the time at end of production). Due to decay of ^177^Lu, the molar activity, total radioactivity, and activity concentration of the [^177^Lu]Lu-DOTA-TATE solution change over time. The product has a shelf life of 72 h from the date and time of calibration. 

## 3. Medicinal and Pharmaceutical Overview

### 3.1. Clinical Indication

The clinical indication of Lutathera^®^ is the treatment of unresectable or metastatic, progressive, well-differentiated (G1 and G2 grade) SSTR-positive gastroenteropancreatic neuroendocrine tumors (GEP-NETs) in adults [15,27]. 

### 3.2. Application

Before starting a treatment with [^177^Lu]Lu-DOTA-TATE, an assessment of the tumor (and metastases) of the patient regarding its/their overexpression of SSTRs should be performed. This can either be done by scintigraphy (e.g., [^111^In]In-DTPA-Octreotide, OctreoScan^®^) or PET (e.g., [^68^Ga]Ga-DOTA-TOC, SomaKit TOC^®^) and it has to be demonstrated that the uptake in tumor tissue is higher than the normal uptake by liver (Krenning score ≥ 2; the Krenning score is a relative uptake score [28]) [15].

In the product monographs and prescribing informations for different countries, the recommended treatment schedule for Lutathera^®^ is described in detail [15,26,27]. The recommended treatment schedule consists of four infusions of 7.4 GBq Lutathera^®^ every eight weeks. The interval between infusions may be prolonged up to 16 weeks when dose-modifying toxicity (concerning the applied radioactivity) occurs. For renal protection, a slow infusion of amino acids (*L*-lysine and *L*-arginine) has to be administered before, during, and after application of the radiopharmaceutical. This infusion may cause nausea and vomiting, due to metabolic acidosis, which may be reduced by antiemetic treatment.

To assess the condition of the patient and, if necessary, to adapt the treatment schedule, a monitoring of the treatment has to be conducted. At least 2–4 weeks and directly before administration of each Lutathera^®^ dose, the liver and kidney function as well as hematological parameters have to be checked. When encountering toxicity, either a prolongation of the treatment interval or an abortion of the treatment may become necessary. 

### 3.3. Pharmacology and Pharmacokinetics

**Figure 2:**
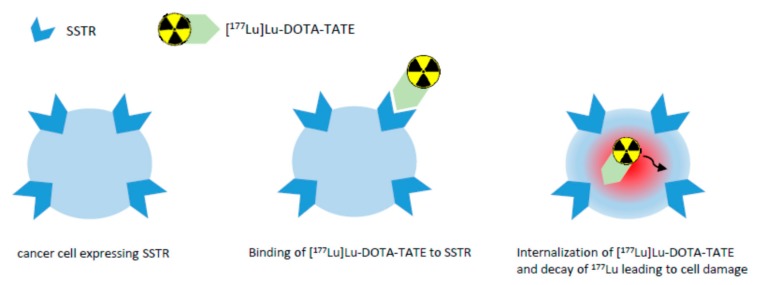
Simplified overview on the mechanism of action of [^177^Lu]Lu-DOTA-TATE.

In a study by Reubi et al. [29], the SSTR subtype affinity profile of a number of somatostatin analogues was investigated, showing, e.g., that DOTA-TATE exhibits high affinity for human SSTR2 (IC_50_ of 1.5 ± 0.4 nM) with much less binding affinity for all other human SSTRs. Complexing this peptide with yttrium resulted in a comparable binding affinity profile and complexing with gallium in an even higher affinity for SSTR2 (IC_50_ of 0.2 ± 0.04 nM). [^177^Lu]Lu-DOTA-TATE shows high binding affinity for SSTR2 as well, and therefore binds to malignant cells with an upregulation of SSTR2 [8]. Upon binding to the receptor, the radiolabeled peptide becomes internalized into the cell and the β particle emission of ^177^Lu induces DNA single- and double-strand breaks, in case of double-strand breaks leading to cell death of the tumor and its SSTR-positive lesions [8]. Figure 2 gives a simplified overview on the mechanism of action of [^177^Lu]Lu-DOTA-TATE. Due to the range of the β particles in comparison to the diameter of a cell, the emitted β particles traverse several cells (10–1000) [30]. This effect has been termed “cross-fire effect” and is responsible for the possible destruction of multiple cells in the vicinity of the tracer-accumulating cell [31]. Using β particle emitters, the cross-fire effect is critical for the improvement of tumor dose homogeneity and for ensuring a sufficient dose for each cell [32]. Another therapeutic effect is the radiation-induced bystander effect: Cells that have not been directly exposed to ionizing radiation, but are in the neighborhood of such cells, behave as if they have been directly hit [31]. Owing to the nanomolar concentration (10 µg/mL; 6.3 × 10^−3^ nM) of the peptide in the sterile injection solution [8], no clinically relevant pharmacodynamic effects are expected.

The pharmacokinetic properties of Lutathera^®^ are described in the product monographs [8,27]. [^177^Lu]Lu-DOTA-TATE is immediately and completely bioavailable through i. v. injection and clears rapidly from blood. The effective half-life in blood in a study with 31 patients was determined to be 0.31 ± 0.13 h [33]. The effective blood elimination half-life is 3.5 ± 1.4 h [26]. Studies using non-radioactive [^175^Lu]Lu-DOTA-TATE showed that protein binding to human plasma is ca. 50% and that transchelation of lutetium to serum proteins is not occurring. Four hours after administration, [^177^Lu]Lu-DOTA-TATE shows uptake in kidneys, tumoral lesions, liver, and spleen, and in some patients, additionally in the pituitary gland and thyroid. Kidney uptake can be decreased by co-administration of amino acids (see above), which reduces the re-absorption of the radiopharmaceutical through the proximal tubules [34]. This is mandatory because the compound is primarily eliminated through the renal system. Data from the Erasmus phase-I/-II and NETTER-1 phase-III trials indicate that ca. 60% of the radiopharmaceutical is eliminated in the urine within 24 h post injection (p. i.) and ca. 65% within 48 h p. i. Metabolism of [^177^Lu]Lu-DOTA-TATE is negligible, as shown by radio-HPLC analysis of urine samples collected 48 h p. i.

Radiation dosimetry of [^177^Lu]Lu-DOTA-TATE has been evaluated in 20 patients in clinical studies [15,27]. The critical organ is the bone marrow. However, with the recommended cumulative dose of 29.6 GBq (4 × 7.4 GBq), neither correlation of hematological toxicity with total administered radioactivity nor with bone marrow absorbed dose has been observed. The kidney is not a critical organ when co-administration of appropriate amino acids is conducted. The median radiation dose to the kidneys can be reduced by 47% (34% to 59%) with co-administration of amino acids [26]. Table 1 summarizes estimated radiation absorbed doses for Lutathera^®^ in the NETTER-1 trial for main organs [26,27].

## 4. Conclusions

Lutathera^®^ ([^177^Lu]Lu-DOTA-TATE) is the first approved radiopharmaceutical for PRRT, a form of endoradiotherapy. This beta-particle emitting radiopharmaceutical has been approved by the FDA, EMA, and Canada Health. It is indicated for the treatment of SSTR-positive GEP-NETs. Combining the radionuclide ^177^Lu with the somatostatin analogue DOTA-TATE, [^177^Lu]Lu-DOTA-TATE delivers ionizing radiation specifically and selectively to tumor cells expressing SSTRs. Therefore, Lutathera^®^ provokes DNA single- and double-strand breaks, in case of double-strand breaks leading to cell death of the tumor and its SSTR-positive lesions. The positive results of the multicenter phase-III clinical trial [16], NETTER-1, comparing the treatment with [^177^Lu]Lu-DOTA-TATE to the treatment with high doses of octreotide LAR, paved the way for its approval by national and international medicines agencies in America and Europe.

## Figures and Tables

**Table 1:** Excerpt of estimated radiation absorbed doses for Lutathera^®^ in the NETTER-1 trial, adapted from [26,27].

Organ	Absorbed Dose Per Unit Activity / Gy/GBq n = 20)		Calculated Absorbed Dose for 4 × 7.4 GBq (29.6 GBq Cumulative Activity) / Gy	
	Mean	SD	Mean	SD
Adrenals	0.037	0.016	1.1	0.5
Breast	0.027	0.016	0.8	0.5
Heart wall	0.032	0.015	0.9	0.4
Kidneys	0.654	0.295	19.4	8.7
Liver *	0.299	0.226	8.9	6.7
Lungs	0.031	0.015	0.9	0.4
Muscle	0.029	0.015	0.8	0.4
Pancreas	0.038	0.016	1.1	0.5
Red Marrow	0.035	0.029	1.0	0.8
Spleen	0.846	0.804	25.1	23.8
Thymus	0.028	0.015	0.8	0.5
Urinary Bladder Wall	0.437	0.176	12.8	5.3
Total Body	0.052	0.027	1.6	0.8

* n = 18.
